# Alcoholic Lung Disease

**Published:** 2008

**Authors:** Corey D. Kershaw, David M. Guidot

**Keywords:** Alcohol abuse, alcoholic lung disorder, pneumonia, acute respiratory distress syndrome (ARDS), acute lung injury, glutathione, alveolar epithelial cell, alveolar macrophage, immune function, CM-CSF treatment

## Abstract

In addition to its well-known association with lung infection (i.e., pneumonia), alcohol abuse now is recognized as an independent factor that increases by three- to four-fold the incidence of the acute respiratory distress syndrome, a severe form of acute lung injury with a mortality rate of 40 to 50 percent. This translates to tens of thousands of excess deaths in the United States each year from alcohol-mediated lung injury, which is comparable to scarring of the liver (i.e., cirrhosis) in terms of alcohol-related mortality. Experimental and clinical studies are shedding light on the basic mechanisms by which alcohol abuse predisposes some people to both acute lung injury and pneumonia. At the same time, novel therapeutic targets could be utilized in treating these uniquely vulnerable people. However, there have been no systems biological approaches to the study of the alcoholic lung to date. This is in part because the association between alcohol abuse and acute lung injury was made relatively recently and remains largely unrecognized, even by lung researchers. In parallel, efforts to study complex diseases such as acute lung injury and pneumonia using a genomics and/or proteomics approach, which involves the study of an organism’s genes and/or proteins, still are in their infancy. However, the alcoholic lung represents a clear example of environment–host interactions that should be well suited for such applications.

Although alcohol abuse has been known for centuries to increase the risk for lung infection (i.e., pneumonia), it only recently has been recognized that alcohol abuse also increases the risk of acute lung injury following major trauma, such as a serious motor vehicle accident, gunshot, or other event requiring hospitalization, or the spread of bacteria attributed to infection (i.e., sepsis) (Moss and Burnham 2003; Moss et al. 1996, 2003). Recent advances in the understanding of alcohol’s effects on both structural and immunological aspects of the lung are bringing to light the precise mechanisms by which alcoholics are predisposed to both pneumonia and acute lung injury.

The well-known acute intoxicating effects of alcohol and the attendant risk of secretions or foreign material entering into the trachea and lungs (i.e., aspiration) are components in the development of alcohol-associated lung (i.e., pulmonary) disease. In the past decade, clinical and experimental evidence has emerged that implicates a chronic damaging chemical imbalance in the cell (i.e., oxidative stress) and consequent cellular dysfunction within the layer of tissue lining the airway (i.e., airway epithelium) as well as pathogen-ingesting white blood cells (i.e., macrophages) in the airway (Bechara et al. 2005; Brown et al. 2001*a*,*b*;Holguin et al. 1998; Kamat et al. 2005). Moreover, now it is recognized that these disruptions in lung function can occur even in young and otherwise healthy individuals long before they develop clinically apparent signs of alcoholic liver disease and/or other end-stage manifestations of longstanding alcohol abuse (Moss et al. 2000). Based on these recent studies, the concept of the alcoholic lung is emerging, which is characterized by severe oxidative stress that alone may not cause detectable lung impairment but may predispose those who are dependent on or abuse alcohol to severe lung injury if they are unfortunate enough to suffer serious trauma or other acute illnesses.

Following a brief overview of the well-known association between alcohol abuse and pneumonia, this article will discuss the newer clinical and experimental evidence linking alcohol abuse and acute lung injury and how systems biological approaches could be applied to identify novel therapeutic targets in the alcoholic lung.

## Alcohol Abuse and Pneumonia

Alcohol abuse has long been recognized as a significant risk factor for pneumonia. The sections below examine the epidemiology of alcohol abuse and pneumonia and the potential mechanisms by which alcohol abuse increases the risk for pneumonia.

### Epidemiology of Alcohol Abuse and Pneumonia

As of 2001, pneumonia was the sixth most common cause of death in the United States, with over 1 million people requiring hospitalization for pneumonia per year (Niederman et al. 1998). In an otherwise healthy person, pneumonia usually has a low mortality and often can be treated without hospitalization with oral antibiotics. However, if hospitalization is required the mortality rises significantly (American Thoracic Society 2001). Further, if patients develop respiratory failure and require care in the intensive care unit (ICU), mortality may exceed 50 percent (American Thoracic Society 2001). Therefore, early identification of patients that may be at higher risk for severe community-acquired pneumonia is important so that clinicians can tailor treatment strategies, such as early ICU admission, to individual patients.

For over a century, alcohol abuse has been well recognized as a significant risk factor for serious pulmonary infections. For example, alcoholic patients are at increased risk for infection with tissue-damaging gram-negative^1^ pathogens, such as *Klebsiella pneumoniae* (Jong et al. 1995), or for the spread of bacteria in the blood (i.e., bacteremia) and shock from typical pathogens, most notably *Streptococcus pneumoniae* (Perlino and Rimland 1985). Importantly, alcoholics also are at increased risk for infections with *Mycobacterium tuberculosis* (Cook 1998).

The impact of alcohol abuse on morbidity and mortality among patients with community-acquired pneumonia is substantial. For example, a study examining the outcomes of alcoholic patients hospitalized for community-acquired pneumonia over a 3-year period (Jong et al. 1995) found that the mortality in this group of patients was 64.3 percent, which was much higher than the predicted death rate for hospitalized patients (approximately 20 percent). An even more alarming result was found in the subset of patients with *Klebsiella pneumoniae* bacteremia. All 11 of these patients died following ICU admission and ventilatory support. A rapidly fatal outcome was noted in this subset, with time from admission to death being 24.6 ± 7.9 hours. Another fatal association between alcohol abuse and pneumonia was identified in a retrospective review of patients admitted with pneumococcal bacteremia that examined a subset with alcoholism and low white blood cell count (i.e., leukopenia) (Perlino and Rimland 1985). Ninety-three patients with pneumococcal bacteremia were identified, 12 of whom had a history of alcohol abuse and a white blood cell count of less than 4,000 cells per cubic millimeter (mm^3^) of blood. Ten of these 12 (83.3 percent) patients died, whereas the mortality in the rest of the cohort was only 22 percent. Overall, these and other studies demonstrate the association between alcohol abuse and community-acquired pneumonia, an association that results in more severe infections and higher mortality.

### Potential Mechanisms by Which Alcohol Abuse Increases Risk for Pneumonia

The mechanisms by which alcohol abuse increases the risk of pneumonia likely are multiple and include increased risk of aspiration of gastric acid and/or microbes from the upper part of the throat (i.e., oropharyngeal flora), decreased mucous-facilitated clearance of bacterial pathogens from the upper airway, and impaired pulmonary host defenses.

Aspiration events are compounded by pathologic changes in the oropharyngeal flora caused by alcohol abuse. For example, the prevalence of oropharyngeal colonization with *K. pneumoniae* may be as much as four times higher in alcoholic compared with nonalcoholic patients (Fuxench-Lopez and Ramierz-Ronda 1978). This increased colonization by pathogenic organisms, combined with the acute intoxicating effects of alcohol and the subsequent depression of the normally protective gag and cough reflexes, leads to more frequent and severe pneumonias from gram-negative organisms. In parallel, defects in the function of the upper airway’s clearance mechanisms in alcoholic patients also play a role. In experimental animal models, alcohol ingestion impairs the function of hair-like projections from cells (i.e., cilia) that sweep mucus out of the lungs, in part by disrupting the normal coordinated ciliary beating that clears pathogens from the airway (Wyatt et al. 2004).

**Figure 1:**
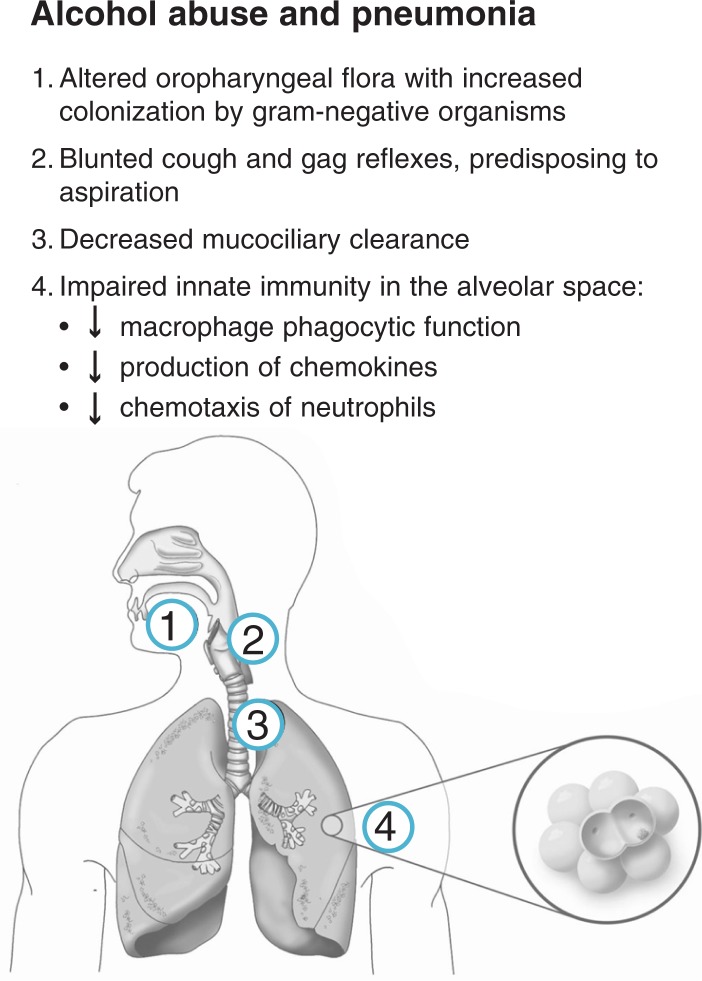
Schematic illustration of the mechanisms by which alcohol abuse increases the risk of pneumonia. In addition to altering the normal microbes from the upper part of the throat (i.e., oropharyngeal flora), alcohol abuse impairs both physical barriers to bacterial entry into the lower airways (by diminishing cough and gag reflexes as well as mucus-facilitated [i.e., mucociliary] clearance from the large airways in the chest) and innate immune barriers to airway pathogens. For example, alcohol abuse impairs pathogen ingestion (i.e., phagocytosis) by white blood cells in the air sacs of the lungs (i.e., alveolar macrophages) and other infection-fighting white blood cells (i.e., neutrophils). NOTE: Aspiration: the entry of secretions or foreign material into the trachea and lungs. Chemokines: small proteins involved in immune function. Chemotaxis: directed movement.

Perhaps the most prominent effects on host defense involve the macrophages present in the air sacs, or alveoli, of the lungs (i.e., alveolar macrophages), the first cellular line of defense against pathogens within the lower airways. In experimental models, chronic alcohol ingestion suppresses the responses of small proteins involved in immune function (i.e., chemokines) as well as the pathogen-clearing and immune responses of alveolar macrophages (Arbabi et al. 1999; Boe et al. 2003; D’Souza et al. 1995; Standiford and Danforth 1997; Zhang et al. 1999, 2002*a*,*b*; Mason et al. 2000) and even increases experimental tuberculosis in mice (Mason et al. 2004). Such controlled laboratory studies support the evolving recognition that alcohol abuse has specific effects on innate immune function within the lower airways and that the increased risk of pneumonia in alcoholics cannot be ascribed solely to factors such as malnutrition, aspiration, or poor oral hygiene. Alcohol-induced defects in alveolar macrophage function and other components of the lung’s innate immune defenses suggest that alcoholics cannot mount an appropriate response to infections, as evidenced by decreased levels of important molecules in the immune system (i.e., interleukins) at the onset of pneumonia and septic shock, a condition that occurs when infection leads to low blood pressure in alcoholics (von Dossow et al. 2004). The combination of impaired innate and adaptive immune responses within the airways is exacerbated by decreased white blood cells (i.e., lymphocytes) within the body’s immune system tissues (i.e., lymphoid tissue) in alcoholics, further stressing an already suboptimal response to infection (Gamble et al. 2006; Happel and Nelson 2005; Nelson and Kolls 2002). Overall, alcohol abuse alters the host immune defenses from the mouth to the alveolar space and increases the risk for bacterial pneumonia as well as tuberculosis. Some of the major mechanisms by which alcohol abuse renders individuals susceptible to pneumonia are illustrated in figure 1.

Recent studies suggest that alcohol ingestion interferes with signaling in the alveolar space by granulocyte/ macrophage colony–stimulating factor^2^ (GM-CSF), which is required for normal innate immune functions in the alveolar macrophage, including ingestion (i.e., phagocytosis) of microbial pathogens (Joshi et al. 2005). This is discussed further in the section that follows.

## Alcohol Abuse and Acute Lung Injury

Alcohol abuse increases the risk for acute lung injury and acute respiratory distress syndrome (ARDS). The epidemiology of alcohol abuse and acute lung injury, the potential mechanisms by which alcohol abuse increases the risk for acute lung injury, and potential treatment strategies are reviewed below.

### Epidemiology of Alcohol Abuse and Acute Lung Injury

ARDS is characterized by a severe deficiency of oxygen in the bloodstream caused by alveolar inflammation (i.e., the accumulation of fluid in the airspaces) in both lungs that cannot be explained by heart failure (i.e., noncardiogenic pulmonary edema). Until recently, ARDS was associated with a very high mortality, ranging from 31 to 74 percent in various studies (ARDS Network [ARDSN] 2000; Ware and Matthay 2000). Unfortunately, despite four decades of laboratory-based and clinical research, no effective pharmacological treatments have been identified. In fact, the only treatment to date that has been shown to decrease ARDS mortality was reported in a National Institutes of Health–sponsored ARDS Network Study in 2000, in which a strategy of low-volume mechanical ventilation (as opposed to the standard volume of ventilation provided) decreased mortality to 31 percent (compared with 40 percent in the conventional treatment group) (ARDSN 2000). Despite implementation of these newer ventilation strategies and earlier recognition of ARDS, some studies suggest that the mortality remains high. For example, a recent prospective^3^ multicenter study in a single geographic location reported an in-hospital mortality of 38.5 percent (Rubenfeld et al. 2005). Therefore, it is important to better understand the pathophysiology of ARDS so that effective treatments can be identified.

Although there are a number of diseases and conditions that can lead to ARDS—pneumonia, sepsis, trauma, and aspiration can account for up to 85 percent of cases (Bernard et al. 1994; Hudson et al. 1995; Ware and Matthay 2000)—only a minority (approximately 30 percent) of these at-risk individuals go on to develop ARDS. For decades there was no explanation as to why some at-risk patients develop the syndrome and others do not. Just over 10 years ago, alcohol abuse emerged as the only independent risk factor known to increase the odds of any given at-risk individual developing ARDS. The first study to identify this association examined 351 critically ill patients at high risk for developing ARDS. The incidence of ARDS in patients with a history of alcohol abuse was 43 percent, compared with an incidence of 22 percent in nonalcoholic subjects (Moss et al. 1996). Further, the in-hospital mortality was 65 percent in alcoholic patients with ARDS, whereas the mortality among nonalcoholic patients was 36 percent. A subsequent study prospectively evaluated 220 patients with septic shock, of whom 30 percent were identified as alcoholics based on responses to the Short Michigan Alcohol Screening Test given to patients and/or their surrogates (Moss et al. 2003). The incidence of ARDS in alcoholic patients was 70 percent compared with 31 percent in nonalcoholic patients. After controlling for potentially confounding variables (such as severity of illness and nutritional indices), the relative risk^4^ of ARDS in alcoholic versus nonalcoholic patients was 3.7:1. That is, alcoholic patients were 3.7 times more likely than nonalcoholic patients to develop ARDS. Overall, 49 percent of those patients who developed ARDS were alcoholics, which is virtually identical to the first study (Moss et al. 1996), in which 51 percent of the patients who developed ARDS were alcoholics.^5^ If these findings are extrapolated to the population at large, then alcohol abuse contributes to the development of ARDS in tens of thousands of patients in the United States each year.

In addition to increasing the risk for developing ARDS, alcohol abuse also makes it more likely that an individual will develop a critical illness that puts them at risk for ARDS in the first place. Alcoholics have increased incidences and death from trauma (Anda et al. 1988), an increased severity of nonpulmonary organ dysfunction in septic shock (Moss et al. 2003), and an increased risk for aspiration (Berkowitz et al. 1973). Alcohol also is a risk factor for the development of scarring of the liver (i.e., cirrhosis), which can lead to increased pressure in the vein that carries blood to the liver (i.e., portal hypertension), and gastrointestinal hemorrhage, which may warrant multiple transfusions of blood products (Hudson et al. 1995), another risk factor for developing ARDS.

### Potential Mechanisms by Which Alcohol Abuse Increases Risk for Acute Lung Injury

Soon after the association between alcohol abuse and ARDS was reported, researchers began to design studies of the mechanisms by which chronic alcohol ingestion increases susceptibility to acute lung injury. Such studies were designed and built on the foundations laid by comparable studies in experimental models of liver injury.

#### Glutathione Depletion

Overwhelming evidence exists for the central role of oxidative stress and depletion of the antioxidant glutathione in the livers of alcohol-fed experimental animals. Glutathione is synthesized primarily by the liver. It is utilized in multiple important pathways, including the detoxification of potentially damaging compounds, facilitation of the excretion of toxic molecules, and control of the induction of proteins involved in inflammation (Kehrer and Lund 1994; Lieber 1993; Morris and Bernard 1994). Glutathione depletion precedes the development of the typical changes in the liver tissue observed with alcohol-mediated liver damage (Lieber 1993). This is supported clinically by the finding that glutathione levels in the liver are decreased in chronic alcoholics regardless of whether there is evidence of cirrhosis (Jewell et al. 1986). In experimental animal models, alcohol administration both decreases glutathione synthesis and increases glutathione oxidation and consumption, thereby severely lowering its levels within liver cells (Lieber 1993). In addition, alcohol inhibits the enzymes with which glutathione interacts to prevent cellular damage (Lieber 1993).

The mechanisms of this decrease in glutathione are under investigation, as are possible ways to increase glutathione levels. Acetaldehyde, a toxic byproduct of alcohol metabolism, may decrease glutathione levels by binding to cysteine, a glutathione precursor, or glutathione itself, although at least one study (Speisky et al. 1988) suggests that the alcohol-induced suppression of liver glutathione levels cannot be completely accounted for by acetaldehyde or alcohol levels alone and that therefore other as-yet-unidentified effects of alcohol metabolism also may contribute to the oxidant stress. In the rat, alcohol intake decreases glutathione levels in the mitochondria of liver cells (Fernandez-Checa et al. 1991; Garcia-Ruiz et al. 1994) through impaired transport of glutathione from the cell’s fluid (i.e., cytosol) to the mitochondria (Fernandez-Checa et al. 1991, 1993). This is a specific toxicity, as other mitochondrial transport functions are preserved (Fernandez-Checa et al. 1993). Subsequent to loss of mitochondrial glutathione, cellular changes occur that are suggestive of acetaldehyde-induced oxidative damage (Fernandez-Checa et al. 1993). Attempts to restore mitochondrial glutathione in the liver cells of alcohol-fed rats with glutathione or the supplement *N*-acetylcysteine (NAC) have been unsuccessful (Fernandez-Checa et al. 1993). However, treatment with either glutathione monoethyl ester (Fernandez-Checa et al. 1991) or a combination of NAC and the glutathione precursor *S*-adenosyl-L-methionine (SAM) (Garcia-Ruiz et al. 1995) increased mitochondrial glutathione and restored liver cell resistance to oxidative injury (Fernandez-Checa et al. 1991). Lieber and colleagues (1990) achieved similar results in a baboon model of cirrhosis.

#### Glutathione and Lung Injury

To study potential mechanisms underlying the association between alcohol abuse and susceptibility to acute lung injury, Holguin and colleagues (1998) utilized a rat model of chronic alcohol ingestion (4 to 6 weeks of ingestion). Consistent with the known effects of alcohol on liver glutathione levels, the researchers first determined that chronic alcohol ingestion in rats dramatically decreases glutathione levels in the alveolar epithelial lining fluid and in the alveolar epithelial type II cells.^6^ In an isolated rat lung, prior chronic alcohol ingestion renders the lung intrinsically susceptible to injury during acute inflammation resulting from exposure to bacteria (Holguin et al. 1998). Specifically, the lung itself is more vulnerable to noncardiogenic pulmonary edema even when isolated from the liver and the systemic circulation (Lois et al. 1999). In parallel, chronic alcohol ingestion impairs surfactant production and increases oxidant-mediated accidental and programmed cell death in alveolar epithelial cells (Holguin et al. 1998; Guidot and Brown 2000; Brown et al. 2001*a*,*b*). Taken together, these initial observations provided the first evidence that chronic alcohol ingestion perturbs the airway epithelium in much the same manner as it renders liver cells and other target cells susceptible to oxidative injury.

Seeking to verify that the relationship between alcohol intake and pulmonary glutathione deficiency in the rat were relevant for humans, Moss and colleagues (2000) measured lung glutathione levels in 19 otherwise healthy alcoholic subjects. Lung glutathione levels in the alcoholic subjects were approximately 80 percent lower than those of nonalcoholic subjects (Moss et al. 2000). These findings, taken together with the findings reported above linking oxidative stress and decreased glutathione in the lungs of alcohol-fed experimental animals, illustrate that the alcoholic lung observed in humans, even in the absence of apparent disease, shows evidence of severe oxidative stress.

Subsequent experimental findings have delineated the complexities of lung glutathione homeostasis and how it is affected by alcohol. Previously, investigators had shown that dietary treatment with glutathione precursors that restore mitochondrial glutathione, such as procysteine or SAM, prevents alcohol-mediated liver disease in experimental animals (Fernandez-Checa et al. 1991; Garcia-Ruiz et al. 1995; Lieber 1993). In contrast, NAC, which for unknown reasons only restores cytosolic glutathione, is less effective at modulating alcohol-mediated liver disease in those models (Fernandez-Checa et al. 1993). Further research has shown that although both NAC and procysteine restore cytosolic glutathione in the type II cells of alcohol-fed rats, only procysteine restores mitochondrial glutathione as well (Brown et al. 2001*a*,*b*; Guidot and Brown 2000). In addition, procysteine, but not NAC, restores surfactant synthesis and secretion by type II cells in vitro (Guidot and Brown 2000), decreases programmed cell death in type II cells in response to inflammatory mediators in vitro (Brown et al. 2001*a*,*b*), restores alveolar epithelial barrier function and lung liquid clearance in vivo (Guidot et al. 2000), and preserves surfactant and decreases respiratory failure during sepsis in vivo (Velasquez et al. 2002).

#### Possible Genetic Factors

As a first step to identify candidate genes that might explain how alcohol-induced oxidative stress renders the lung susceptible to acute lung injury, the authors compared lungs from control- and alcohol-fed rats using genetic analysis. Although hundreds of genes differed between the control- and alcohol-fed rats, the authors noted with interest that the expression of angiotensinogen and angiotensin converting enzyme (ACE), which are key components of the renin-angiotensin system,^7^ were markedly increased in the alcohol-fed rats, and this guided subsequent studies that implicated this pathway in alcohol-induced oxidant stress in our experimental models (Bechara et al. 2003, 2004; Pliikandriotis et al. 2006). In addition, the expression of a protein involved in immune system regulation, transforming growth factor β_1_ (TGFβ_1_), also was markedly increased in the alcohol-fed rat lungs (Bechara et al. 2004).

- ***Angiotensin.*** Chronic alcohol ingestion increases levels of an agent involved in narrowing blood vessels (i.e., angiotensin II) in rodents (Wright et al. 1986) and humans (Linkola et al. 1979; Puddey et al. 1987), and it has been postulated that activation of the renin-angiotensin system may explain the association between alcohol abuse and hypertension in humans (Wigle et al. 1993). However, mice deficient in ACE, which would therefore have less angiotensin, have less lung injury following acid aspiration or sepsis (Imai et al. 2005), and at least one clinical study suggests that people who do not express the ACE gene variant associated with increased enzyme activity are at lower risk of ARDS (Marshall et al. 2002).
- ***TGFβ****_1_*. Recent experimental evidence suggests a role for TGFβ_1_ as a mediator of acute lung injury (Pittet et al. 2001). TGFβ_1_ has multiple potential effects on tissue injury and repair during lung injury. It appears to mediate cell death (Hartsough and Mulder 1997), glutathione depletion (Arsalane et al. 1997), and disruption of epithelial integrity (Hartsough and Mulder 1997; Pittet et al. 2001) in experimental models. These effects parallel the authors’ own findings in the lungs of alcohol-fed rats. Importantly, angiotensin II is well known to induce TGFβ_1_ expression in other tissues such as the kidney (Gilbert et al. 1998; Junaid et al. 1997; Noble and Border 1997). Therefore, the authors set out to determine the role(s) of the renin-angiotensin system, particularly angiotensin II, and TGFβ_1_ in experimental models of alcohol-induced susceptibility to acute lung injury.

#### Roles of Angiotensin II and TGF in Lung Injury

The mechanisms by which alcohol ingestion causes such profound oxidative stress and cellular dysfunction within the alveolar space now are becoming apparent. Consistent with the gene expression data reported above, the major culprit appears to be aberrant activation of the renin-angiotensin system and the subsequent actions of angiotensin II within the lung itself. Specifically, alcohol ingestion, via angiotensin II, activates activity of the enzyme NADPH oxidase within the lung, which in turn increases the production of highly reactive and damaging free radicals known as reactive oxygen species (Polikandriotis et al. 2006). Further, when alcohol-fed rats have their diets supplemented with an inhibitor of angiotensin II formation or a blocker of the molecule that binds angiotensin type 1 (AT_1_) (i.e., AT_1_ receptor), their lungs are completely protected from glutathione deficiency and increased susceptibility to injury from toxins produced by bacteria (Bechara et al. 2005).

Angiotensin II activity in the alcoholic lung also causes a shift in angiotensin II receptor expression in the alveolar epithelium, such that the type 2 receptor (i.e., AT_2_ receptor) becomes predominant (Bechara et al. 2003). This change renders the epithelial cells susceptible to programmed cell death. This may reflect a compensatory response to excess AT_1_ activation during chronic alcohol ingestion.

**Figure 2:**
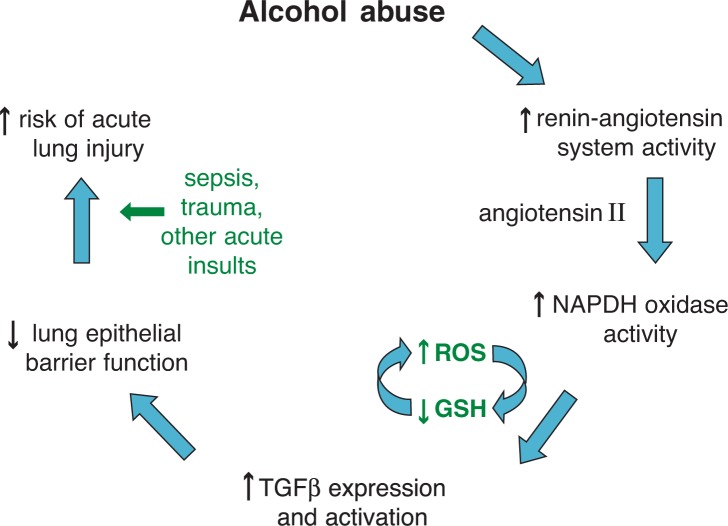
Proposed pathophysiological sequence by which alcohol abuse renders the lung susceptible during acute inflammatory stresses such as infection (i.e., sepsis) and trauma. Alcohol abuse activates the renin-angiotensin system^1^ which, via its active mediator angiotensin II, increases the expression and activity of the enzyme NADPH oxidase, a potent generator of highly reactive and damaging free radicals known as reactive oxygen species (ROS) such as superoxide anion and hydrogen peroxide. In parallel to, and perhaps as a consequence of, the increased ROS production, the levels of the protective antioxidant, glutathione (GSH), are decreased by as much as 80 to 90 percent in the air sacs of the lung (i.e., alveoli). As a consequence, the expression of a protein involved in immune system regulation, transforming growth factor β (TGFβ), is increased. When activated in the alveoli (particularly during acute inflammatory stresses), TGFβ disrupts the normally tight alveolar epithelial barrier that allows the alveoli to remain air-filled. The net result is a marked increase in the leakage of protein and fluid into the alveolar space and the development of respiratory failure. ^1^ The renin-angiotensin system is a hormone system that helps regulate long-term blood pressure and extracellular volume in the body.

As noted previously, alcohol-induced oxidative stress impairs multiple critical cellular functions within the lung. In particular, the critical barrier function within the alveolar epithelium is compromised. Under normal conditions, the alveolar epithelium is a tight barrier that allows the alveoli to remain air filled despite their close proximity to the lung’s small blood vessels (i.e., capillaries), through which the entire cardiac output courses. This dynamic barrier physically restricts the leakage of fluid into the alveolar space but also actively transports sodium and fluid out of the alveolar space in order to maintain this gas exchange unit. In light of the effects of alcohol on alveolar epithelial viability reported above, it is not surprising that chronic alcohol ingestion increases alveolar epithelial protein leakage and decreases the lungs’ ability to remove liquid in the rat model in vivo (Guidot et al. 2000). Again consistent with the gene expression data reported above, recent findings suggest that TGFβ_1_ mediates many of these effects. Chronic alcohol ingestion, via the sequential actions of angiotensin II and glutathione depletion, markedly increases the expression of TGFβ_1_ in the rat lung (Bechara et al. 2004, 2005). During acute inflammatory stresses such as sepsis and trauma, TGFβ_1_ is released and activated in the alveolar space, where it can cause the alveolar epithelial barrier dysfunction described above (Bechara et al. 2004). Therefore, the experimental findings to date implicate the pathophysiological sequence in the alcoholic lung shown in figure 2.

## Potential Treatment Strategies

The findings reported here reflect how much has been learned in the past decade since it was first recognized that alcohol abuse increases the risk of ARDS, even in young and previously healthy people. The extensive research in this area suggests that although glutathione deficiency is a useful marker of severe structural and functional abnormalities in the alcoholic lung, treatment strategies necessary to modify the risk of lung injury will require more than glutathione replacement alone. Whereas oxidative stress is more directly involved in causing the pathophysiology of the alcoholic lung, the susceptibility to injury reflects cellular damage that cannot be quickly reversed with glutathione replacement alone. This is complicated by the fact that there are no feasible strategies to specifically block the actions of TGFβ_1_ in the clinical setting. Therefore, the current challenge is to identify other mechanisms of alcohol-mediated oxidative injury that are amenable to therapeutic intervention.

An intriguing candidate for just such an intervention is GM-CSF. As mentioned above, Joshi and colleagues (2005) recently demonstrated that chronic alcohol ingestion impairs GM-CSF–dependent alveolar epithelial cell and macrophage function. These discoveries were initiated by the finding that, when delivered via the upper airway, GM-CSF restored alveolar epithelial barrier function and fluid transport in alcohol-fed rats, even when bacteria-released toxins were present in the blood (Pelaez et al. 2004). Joshi et al. (2005, 2006) subsequently found that chronic alcohol ingestion decreases the expression of GM-CSF receptors in the airway epithelium and macrophages and, in turn, dampens intracellular signaling to the protein responsible for GM-CSF gene expression. As a consequence, GM-CSF–dependent functions in each cell type are impaired. Although the mechanisms remain to be delineated, treatment with recombinant GM-CSF restores GM-CSF receptor expression and signaling and normalizes both alveolar epithelial barrier function (Joshi et al. 2006) and alveolar macrophage immune function (Joshi et al. 2005). GM-CSF treatment is widely used to improve bone marrow recovery following chemotherapy for malignancies. However, the major site of GM-CSF production actually is in the airway epithelium, where its actions are partially blocked by alcohol abuse. Research has evaluated GM-CSF treatment for acute lung injury. A clinical trial of 18 patients with septic shock demonstrated that patients who received recombinant GM-CSF treatment had less severe lung injury than those who received a placebo (Presneill et al. 2002). Presently, this strategy has not been expanded into a larger clinical trial. However, it is exciting to consider that GM-CSF treatment might be efficacious in alcoholic individuals with acute illnesses such as trauma or sepsis, in which the known incidence of ARDS is approximately 70 percent. Such a strategy also might augment alveolar macrophage immune functions and improve outcomes in alcoholic subjects with severe community- or hospital-acquired pneumonia, as the aforementioned experimental findings suggest that alveolar macrophage immune function in the alcoholic lung also is rapidly restored by recombinant GM-CSF treatment (Joshi et al. 2005).

**Figure 3:**
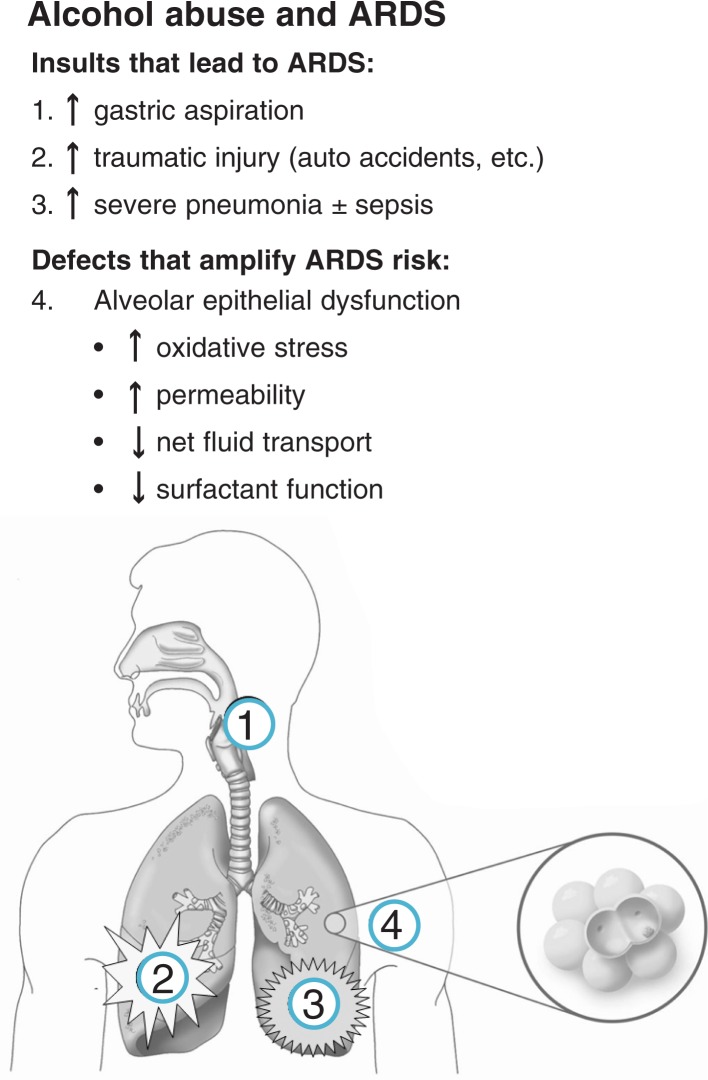
Schematic illustration of the mechanisms by which alcohol abuse increases the risk of the acute respiratory distress syndrome (ARDS). In addition to increasing the risk of developing acute illnesses that lead to ARDS, such as the entry of stomach secretions into the trachea and lungs (i.e., gastric aspiration), trauma, and severe pneumonia, alcohol abuse impairs the function of the lining of the lung’s air sacs (i.e., alveolar epithelium), thereby rendering the lung susceptible to injury from fluid accumulation (i.e., ARDS) that might not otherwise occur in a healthy person who experiences the same initial injury or infection. NOTE: Oxidative stress: damaging chemical imbalance in the cell. Surfactant: the substance that serves to maintain the stability of lung tissue by reducing the surface tension of fluids that coat the lung.

In summary, in addition to its well-known association with pneumonia, alcohol abuse independently increases the risk of ARDS two- to four-fold in at-risk individuals, and this is exacerbated by the fact that alcohol abuse also increases the risk for trauma, sepsis, and other acute illnesses that lead to ARDS. Although the pathological mechanisms by which alcohol abuse renders the lung susceptible to acute injury from fluid accumulation likely reflect multiple cellular functions within the lung, defects in the alveolar epithelium play a major role. In concert with these defects in alveolar epithelial function, alcohol-mediated suppression of alveolar macrophage immune functions are central to the increased risk for pneumonia. figure 3 shows a highly simplified illustration of how alcohol abuse increases the incidence of ARDS, by augmenting the chances of developing an at-risk diagnosis such as trauma and by impairing critical functions within the alveolar epithelium.

## Systems Biology and the Study of Alcoholic Lung Disease

As discussed in this review, genetic analysis has helped to identify potential candidate genes involved in alcohol-induced lung dysfunction that might explain the newly identified association between alcohol abuse and acute lung injury in humans. Although several genes of interest were identified and pursued as has been discussed, the vast majority of the genes that displayed significantly altered expression in the alcohol-fed rat lung have not yet been evaluated. In fact, the full power of genomic and proteomic tools, which are used to study an organism’s genes and/or proteins, only now are being applied to complex lung diseases. A recent volume of the *Proceedings of the American Thoracic Society* (Volume 4, Number 1, January 2007) was devoted entirely to a virtual symposium entitled “Making Genomics Functional in Lung Disease.” Included in the many provocative articles in this special issue were discussions about the application of genomic techniques to identify candidate genes in diverse pulmonary diseases, including acute lung injury (Meyer and Garcia 2007), pulmonary fibrosis (Studer and Kaminski 2007), the development of small inflammatory nodules (i.e., sarcoidosis) (Iannuzzi and Rybicki 2007), and lung cancer (Borczuk and Powell 2007). No known research has applied such approaches to the evaluation of the alcoholic lung in humans, but there is great promise that the rapidly evolving tools of systems biology will accelerate the pace at which researchers are discovering how alcohol abuse produces such devastating lung damage.

## Footnotes

1 Gram-negative bacteria are a common class of bacteria normally found in the gastrointestinal tract that can be responsible for disease. Bacteria are considered to be gram-negative because of their characteristic staining properties under the microscope.

2 Granulocyte/macrophage colony–stimulating factor (GM-CSF) is a protein involved in the immune response. It stimulates the production of macrophages and another type of white blood cell known as granulocytes.

3 In a prospective study, participants are identified and then followed forward in time.

4 Relative risk is the risk of an event (or of developing a disease) relative to exposure. Relative risk is a ratio of the probability of the event occurring in the exposed group versus the control (nonexposed) group.

5 In the original study, the incidence of ARDS in alcoholics was 43 percent. Of all the patients who developed ARDS, 51 percent were alcoholics, compared with 49 percent in the second study.

6 Alveolar epithelial type II cells synthesize, secrete, and recycle all components of the substance (i.e., surfactant) that serves to maintain the stability of pulmonary tissue by reducing the surface tension of fluids that coat the lung.

7 The renin-angiotensin system is a hormone system that helps regulate long-term blood pressure and extracellular volume in the body.
